# Trough Concentrations of Specific Antibodies in Primary Immunodeficiency Patients Receiving Intravenous Immunoglobulin Replacement Therapy

**DOI:** 10.3390/jcm10040592

**Published:** 2021-02-04

**Authors:** Ori Hassin, Yahya Abu Freih, Ran Hazan, Atar Lev, Keren S. Zrihen, Raz Somech, Arnon Broides, Amit Nahum

**Affiliations:** 1 Pediatrics Department A, Soroka University Medical Center, 8410101 Beer-Sheva, Israel; orihasin@gmail.com (O.H.); ranranha@gmail.com (R.H.); 2 The Primary Immunodeficiency Research Laboratory, The Shraga Segal Department of Microbiology, Immunology and Genetics, Faculty of Health Sciences, Ben-Gurion University of the Negev, 8410101 Beer-Sheva, Israel; yahyaa@post.bgu.ac.il; 3 Jeffrey Modell Foundation Center, Sheba Medical Center, Pediatric Department A and Immunology Service, Edmond and Lily Safra Children’s Hospital, 5262000 Tel HaShomer, Israel; Atar.Lev@sheba.health.gov (A.L.); kerenzrien@gmail.com (K.S.Z.); Raz.Somech@sheba.health.gov.il (R.S.); 4 Sackler Faculty of Medicine, Tel Aviv University, 6990871 Tel Aviv, Israel; 5 Immunology Clinic, Soroka University Medical Center, and Faculty of Health Sciences, Ben-Gurion University of the Negev, 8410101 Beer-Sheva, Israel; broides@bgu.ac.il

**Keywords:** intravenous immunoglobulin, specific antibody, trough concentration, immunodeficiency

## Abstract

Immunoglobulin replacement therapy is a mainstay therapy for patients with primary immunodeficiency (PID). The content of these preparations was studied extensively. Nevertheless, data regarding the effective specific antibodies content (especially in the nadir period), and, in different groups of PID patients is limited. We studied trough IgG concentrations as well as anti-Pneumococcus, anti-Haemophilus influenzae b, anti-Tetanus, and anti-Measles antibody concentrations in 17 PID patients receiving intravenous immunoglobulin (IVIg) compared with healthy controls matched for age and ethnicity. We also analyzed these results according to the specific PID diagnosis: X-linked agammaglobulinemia (XLA), combined immunodeficiency (CID), and ataxia telangiectasia (AT). We recorded a higher concentration of anti-pneumococcal polysaccharide antibodies in healthy controls compared to the entire group of PID patients. We also found significantly higher anti-tetanus toxoid antibody concentrations in the XLA patients, compared to CID patients. Anti-Haemophilus Influenzae b antibody titers were overall similar between all the groups. Interestingly, there were overall low titers of anti-Measles antibodies below protective cutoff antibody concentrations in most patients as well as in healthy controls. We conclude that relying on total IgG trough levels is not necessarily a reflection of effective specific antibodies in the patient’s serum. This is especially relevant to CID patients who may have production of nonspecific antibodies. In such patients, a higher target trough IgG concentration should be considered. Another aspect worth considering is that the use of plasma from adult donors with a waning immunity for certain pathogens probably affects the concentrations of specific antibodies in IVIg preparations.

## 1. Introduction

Intravenous immunoglobulins (IVIg) replacement is a mainstay therapy for patients with primary immunodeficiency, and particularly for patients with defects in B or combined B and T cell dysfunction. B cell defects, such as X-linked Agammaglobulinemia (XLA) in which immunoglobulins production is usually severely impaired and Common Variable Immunodeficiency (CVID), where B cells produce some immunoglobulins but lack the ability to respond to specific stimuli, comprise the majority of patients in need for IVIg worldwide [1]. Another significant group of patients requiring immunoglobulin replacement are patients with combined immunodeficiency, such as patients with what can be considered as “hyper IgE syndromes” (DOCK8 deficiency, STAT3 deficiency, IL6 receptor deficiency), hyper IgM syndrome, ataxia telangiectasia patients, and “leaky SCIDs” such as hypomorphic presentations of artemis, as well as others [2,3,4,5,6].

Since its’ introduction, IVIg treatment has had an enormous impact on both the survival and quality of life of these patients. The preparation of IVIg is regulated and agencies such as the World health Organization (WHO) have issued guidelines for manufacturing, including a minimum number of blood donor plasma to be included in each batch. This was decided in order to give the patients a wide variety of antibodies, protecting against as many pathogens as possible, with a wide repertoire of antibodies [7]. Indeed, many studies have focused on the content of IVIg preparations and most of them found no major difference between preparations and adequate concentrations of antibodies in commercial IVIg products. Such was the case for Pneumoccocal polysaccharides antibodies [8], antibodies against hepatitis B and A [9], Enterovirus antibodies [10], as well as Haemophilus influenzae type b (Hib), and, Niesseria meningitides antibodies [11]. 

There are a few studies assessing the titers of specific immunoglobulins in patients receiving immunoglobulins, in both intravenous and subcutaneous forms. These studies focused on concentrations of pneumococcal anti polysaccharides antibodies to 23 valent vaccine serotypes, and antibodies against Hib, with a recent study measuring antibodies concentration against polio virus [12,13,14].

In the current study we were interested in a specific point in time—the last week before a routine IVIg transfusion—which represents the time period when our patients are most vulnerable to infections. Therefore, in this study we compared the trough concentrations of specific antibodies in the patient’s serum and not in the IVIg preparation. A second question we attempted to answer is whether we could find differences in specific antibodies titers between different groups of patients. Some of the patients with combined immunodeficiency produced immunoglobulins on their own, (although not effective), while for most patients with X-linked agammaglobulinemia (XLA), IgG was entirely derived from the IVIg product. As a clinical routine we monitored total IgG trough levels comprised of both the patients’ own production of IgG in combination with the infused dose. Clinicians were careful not to reach extremely high concentrations of immunoglobulins so as not to cause hyper-coagulability. Alas, the effective specific antibodies titers in patients in whom non-specific immunoglobulin production occurs may be lower than those found in patients without self-produced IgG, and, if this is indeed the case it should be taken into consideration upon determining the individual patient desired trough and dose. 

## 2. Methods

### 2.1. Patients 

The study was approved by the institutional research ethics board (study # 0212-17-SOR). Blood was drawn after informed consent was obtained from the parents or legal guardian. 

The study population consisted of 17 patients aged 1–18 years old treated at Soroka University Medical Center and a group of 17 healthy individuals matched for age and ethnicity. All patients were receiving uninterrupted IVIg as replacement therapy during a minimum of 6 consecutive months, in regular time intervals (for most patients; 4 weeks, and for 2 patients; every 3 weeks), with the same commercial preparation. We have excluded from the study patients with uncontrolled inflammation, chronic inflammatory diseases or catabolic states that would cause unusual consumption or loss of antibodies. 

Control serums were drawn from healthy patients who were hospitalized for elective minor surgery or admitted to non-inflammatory and non-infectious diseases. We have reviewed vaccination records from electronic health records and/or the vaccine hardcopy log book of our controls to verify adherence to vaccination schedule with attention to all those relevant to the antibodies measured in our study. 

### 2.2. Sample Collection and Handling

Blood samples were collected before infusion of IVIg. Serum from whole blood was separated by centrifugation at 1200× *g* rpm for 5 min, aliquoted, and frozen immediately at −70 °C for later analysis. 

### 2.3. Determination of Specific Antibodies Levels

The serum was tested in duplicates, using ELISA kits for detection of the different specific antibodies against pathogens. Anti-pneumococcal VaccZyme IgG PCP kit, VaccZyme Tetanus toxoid IgG kit, and VaccZyme *Haemophilus influenzae* type b IgG kit, (The Binding Site, Birmingham, UK), and Human Anti-Measles IgG ELISA kit (#108750 Abacam, Cambridge, MA, USA). Samples were tested in duplicates and in cases with discrepancies the sample was tested again. Assays included internal control and carried according to the manufacturer protocols.

### 2.4. Statistics 

Analyses of continuous variables were parametric using student t-test. Non-parametric Mann–Whitney test was used if parametric assumptions could not be satisfied. All statistical tests and/or confidence intervals, as appropriate, were performed at α = 0.05 (2-sided). *p*-values reported were rounded to three decimal places. The data was analyzed using GraphPad Prism 6.01.

## 3. Results

### 3.1. Patients Epidemiologic Data and Total IgG Levels 

We enrolled a total of 17 patients with primary immunodeficiency treated with IVIg in our institute and 17 healthy control subjects. 

Overall, the ages and ethnicity of our control and patient populations were similar, with a statistically non-significant younger median age of 8.6 years in controls compared to 11.9 years in the patients (Table 1). 

**Figure 1:**
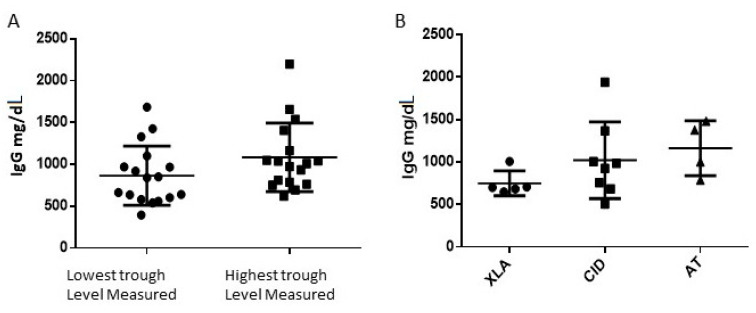
Mean total IgG concentration measured in patients during consecutive 6 months. (**A**). lowest and highest trough levels in our patients. *p* value = 0.088; circle symbols = lowest trough IgG level measured in patients; square symbols = highest trough level measured in the serum of patients. (**B**). IgG trough level median of 6 consecutive months in three groups of patients X-linked agammaglobulinemia (XLA) Combined immunodeficiency (CID) Ataxia Telangiectasia (AT), *p* value = 0.163; circle symbols = group of patients diagnosed with X-linked agammaglobulinemia; square symbols = group of patients with combined immunodeficiency; triangle symbols = group of patients diagnosed with Ataxia Telangiectasia.

All patients were treated with IVIg for at least 6 months without interruption. During this period patients received the same commercial product, either A (11 patients) or B (6 patients) for the entire period. Patients who required switching to a different brand for any reason were not included in the study. The IgG trough levels were all above those recommended for age during the entire period, and, without any significant variation during this period as reflected from means of minimal and maximal levels measured in each patient (Figure 1A). Healthy controls received vaccinations as recommended by the health ministry including HBV, HAV, DTPHib, IPV, PCV13, and MMRV.

The study included five patients with X-linked agammaglobulinemia (XLA), four patients with Ataxia Telangiectasia (AT), and eight patients with various combined immunodeficiency (CID) diseases; STAT3 deficiency, CD40 ligand deficiency and IL6 receptor deficiency. Four patients carried a clinical diagnosis of CVID without a definite genetic diagnosis. Median trough IgG was somewhat higher in the CID and AT groups in comparison to the XLA patients, but this difference did not reach any statistical significance (*p* value = 0.24) (Figure 1B).

### 3.2. Specific Antibodies Trough Levels in the Entire Group of Patients

**Figure 2:**
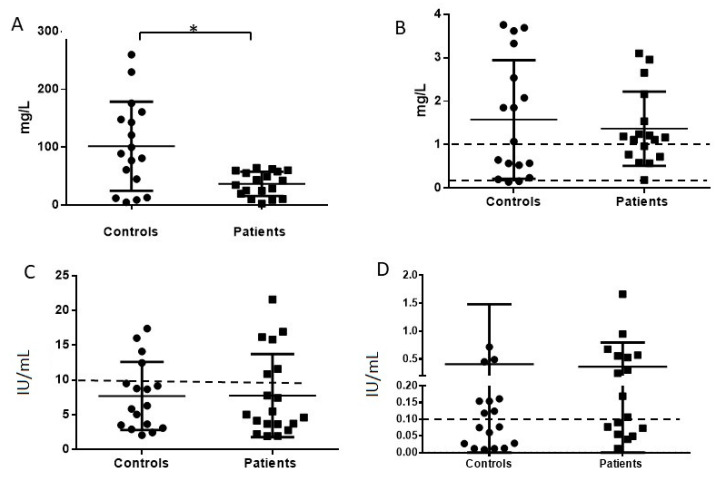
Specific antibodies titer in healthy controls and the entire cohort of patients treated with IVIg (**A**). Pneumococcal anti polysaccharide * *p* value = 0.0015, (**B**). Haemophilus Influenza B antibodies titer. Dashed lines are marked at short term immunization titer and optimal long-term level at 0.15 mg/L and 1 mg/L. (**C**). Measles IgG antibodies titer, Assay cutoff value at 10 IU/mL. *p* value = 0.65 (**D**). Tetanus toxoid antibodies titer in healthy controls and entire patient cohort. Dashed line marks cutoff value at the 0.1 IU/mL considered protective. *p* value = 0.186; circle symbols = healthy control subjects; square symbols = immunodeficiency patients.

We have tested several specific immunoglobulins titers which represent some of the common pathogens and those which the population is vaccinated against. Anti-Pneumococcal polysaccharides antibodies were measured as total of the 23 serotypes using a clinically valid assay. Measured titers revealed a clearly significant difference between healthy controls and the entire group of patients, with a *p* value of 0.0015, (Figure 2A). As for anti-Hib antibodies, most healthy controls and patients had titers above 0.15 mg/L which is considered as an acceptable short term protective level, and, as many as 2/3 of patients and just over half the controls had a level of 1 mg/L and above, which is an optimal long term protective level as considered by WHO (WHO-Haemophilus influenzae type b (Hib) Vaccination Position Paper—July 2013) [15], (Figure 2B).

Anti-Measles antibody concentrations in our controls and patients were found to be lower than the assay’s protective cutoff of 10 IU/mL concentrations in the majority of the individuals tested. Only four in each group had titers which were clearly positive (two additional patients had titers just above cutoff), (Figure 2C). Anti-Tetanus toxoid antibodies concentrations in both controls and PID patient groups were extremely variable. When considering the cutoff level as protective titer at 0.1 IU/mL, only 8 of the 17 healthy subjects and 10 of 17 patients had these concentrations, (Figure 2D).

### 3.3. Specific Antibodies Trough Levels by Diagnosis of Primary Immunodeficiency 

**Figure 3:**
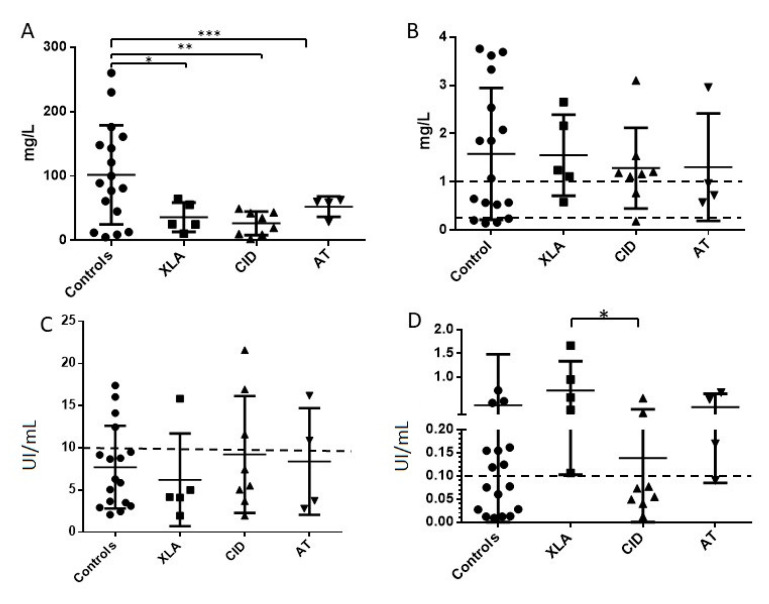
(**A**). Pneumococcal anti polysaccharide antibodies titer in healthy controls and groups of patients treated with IVIg * *p* value = 0.0058, ** *p* value = 0.0011, *** *p* value = 0.025 (**B**). Haemophilus Influenza B antibodies titer in healthy controls and groups of patients treated with IVIg. Dashed lines are marked at cutoff levels for short term immunization titer and optimal long-term level at 0.15 mg/L and 1 mg/L. (**C**). Anti-Measles IgG antibodies titer in healthy controls and groups of patients treated with IVIg, assay positive cutoff value at 10 IU/mL. *p* value = 0.814 (**D**). Tetanus toxoid antibodies titer in healthy controls and groups of patients treated with IVIg. Dashed line marks cutoff value at the 0.1 IU/mL considered protective. * *p* value = 0.027; circle symbols = healthy control subjects; square symbols = patients diagnosed with X-linked agammaglobulinemia; triangle symbols (pointing upward) = patients with combined immunodeficiency; triangle symbols (pointing downward) = patients diagnosed with Ataxia Telangiectasia.

When looking at specific antibody titers in various groups of patients we could identify some differences between them. In the healthy control group the mean value for anti- pneumococcal anti polysaccharides titer was 100 mg/mL, whereas in XLA patients the mean was 36.1 mg/mL in, a mean of 26.58 mg/mL in CID patients and 58 mg/mL in the AT group of patients (Figure 3A). These differences were significant with *p* values between Control, and each of the patient groups, *p* value = 0.0058, *p* value = 0.0011 and *p* value = 0.025 for XLA, CID, and AT groups respectively. Yet, it is important to note that the acceptable protective concentrations were attained in all groups.

Nonetheless, this was not the case for anti-Tetanus toxoid antibody titers. There was no significant difference between means of healthy controls’ serum levels to those found in the entire patient group (*p* value = 0.683) (Figure 2D). However this was not the case when examining the various groups of PID patients: XLA patients had the highest mean concentration (0.7 IU/mL) compared to CID patients with a mean concentration of 0.13 IU/mL (*p* value of 0.027). In the latter group of patients, the mean level was just below the 0.1 IU/mL cutoff, which is considered as a protective concentration. Most of the patients in this group showed sub-optimal titers. The small group of AT patients had a mean titer of 0.365 IU/mL and all patients had levels above the cutoff (Figure 3D).

As for anti-HiB antibody concentrations, XLA patients had a similar mean (1.55 mg/L) to healthy controls, which was 1.57 mg/L and higher than both CID (1.28 mg/L) and AT patients (1.3 mg/L). These differences did not reach statistical significance (Figure 3B). 

Analysis of anti-Measles antibody concentrations according to groups of patients did not show significant differences between the groups (*p* value = 0.819) and as mentioned above most patients did not reach the protective cutoff concentrations in our assay in most of the patients and controls (Figure 3C). 

## 4. Discussion

Immunoglobulins replacement therapy is a mainstay tool in our efforts to prevent infections in primary immunodeficiency patients. Previous work by several groups studied the presence of different specific antibodies in almost all commercially available IVIg preparations, among them those used in our institute. Most of these studies showed that specific antibodies concentrations in the preparations were adequate and in agreement with current guidelines [8,9,10]. While we are constantly monitoring total IgG trough levels, as part of our routine assessment of patients receiving IVIG, the concentrations of specific protective antibodies against pathogens are not routinely reviewed. The last several days prior to the scheduled IVIg infusion is the time period when our patients are most vulnerable and are at risk for infections. The present study shows the concentrations of specific antibodies at this unique point in time, revealing some interesting findings. 

Testing the specific IgG antibodies against anti-Pneumococcal Capsular Polysaccharides (PCP), revealed that the entire cohort of immunodeficiency patients had significantly lower concentrations of antibodies than the healthy control group. This is probably due to the fact that healthy children are frequently exposed to pneumococci and are constantly producing and maintaining a relatively high concentration of specific antibodies during their childhood years. On the contrary, adults are less exposed to pneumococci—they are the source for plasma derived IVIg products—which may explain the lower titers found in patients’ serum receiving IVIg. These results should be viewed with the known limitations of PPS testing in the era of conjugated vaccines which can increase the titer of the vaccine serotypes by producing a T cell mediated response rather the classic T independent response to polysaccharides. This could possibly mislead us in thinking our subject has an adequate level of pneumococcal antibodies whereas there could be a complete lack of antibodies against non-vaccine serotypes [16,17]. Unfortunately, we do not have in our country any clinical facility for specific serotype testing, and, as far as we know the only commercial kit is no longer available. 

Anti-Measles specific antibody titers were uniformly poor in both immunodeficiency patients and healthy controls. In the healthy control group, slightly over half of the subjects and as many as 2/3 of the patients had titers considered negative. This finding is alarming especially after the reemergence of measles in recent years [18]. As suggested from population studies, this is probably a reflection of the waning of immunity to this vaccine, and perhaps a lack of boosting in the adult population [19,20]. Measles is a severe threat in patients with primary immunodeficiency, who are at risk to develop both short and long term complications, such as severe pneumonia and encephalitis [21]. This result should alert us in regards to our patients and the use of an effective preparation as a preventive measure if needed. Anti-HiB antibody mean titers were very similar between control and patient groups, however, 1/3 of our patients did not have a long term protective optimal titer in their serum. The measured titer in healthy controls—with their intact immune memory—is probably sufficient, whereas, this very same titer could potentially be inadequate for our patients. Interestingly, one of our patients did experience Hib meningitis while on IVIg treatment. Although one cannot draw conclusions from one event, it is worthwhile thinking about the adequate protective concentrations of specific antibodies in immunodeficient patients receiving IVIG replacement therapy. 

We have further analyzed and dissected our results to different groups of patients. The rationale was that some of the CID patients may have their own production of antibodies, and so, the apparent trough of IgG reflects the sum of self, and administered IVIg. In CID patients in whom the IgG production results in ineffective IgG, this potentially may lower the concentration of effective specific antibodies. We have shown, at least for Tetanus toxoid antibodies, that patients with X-linked agammaglobulinemia (in which all IgG is derived from the IVIg product) had significantly higher concentrations than patients with combined immunodeficiency with the same or even higher total, non-specific IgG trough levels. This is somewhat worrisome, as among the group of CID patients, six out of eight showed non protective levels of tetanus antibodies. As for Pneumococcal polysaccharides antibodies, both XLA and AT patients had somewhat higher levels than CID patients, but differences were not statistically significant. Our study bares some limitations, the major one being the small number of patients in each group of PID patients. This therefore makes the statistical analysis more difficult to interpret. This could be overcome by larger scale studies with more patients from different locations. Another limitation is our limited ability to assess the function of such antibodies. Such is the case for pneumococcal antibodies, where assays evaluating the neutralizing ability of antibodies was studied [16].

The data presented here may possibly lead to re-evaluation of the regimens used for IVIg dosing for certain groups of primary immunodeficiency patients. It is possible that trough levels of specific immunoglobulins could be used as an adjunct parameter when deciding on the appropriate IVIg dose, and that patients who are producing their own antibodies should receive IVIg in a dose that is not solely based on their total IgG trough level. 

## Figures and Tables

**Table 1:** Summary of epidemiologic and demographic data of patients and control group.

Characteristic	PID Patients	Control Group (Non-PID)
Number	17	17
Median age (range)	11.9 (1.1–17.9)	8.6 (1.1–18)
Gender No. (%)	Male = 15 (88.2%) Female = 2 (11.8%)	Male = 12 (70.5%) Female = 5 (29.5%)
Ethnicity	Jewish = 3 (17.6%) Bedouin = 14 (82.4%)	Jewish = 1 (5.8%) Bedouin = 16 (94.2%)
Groups of PID patients No. (%)	CID = 8 (47%) XLA = 5 (29.4%) AT = 4 (23.6%)	

PID—primary immunodeficiency, XLA—X-linked agammaglobulinemia, AT—Ataxia Telangiectasia, CID—Combined Immunodeficiency.

## Data Availability

All supporting data is available on request from corresponding author.
